# Involvement of soluble scavenger receptor A in suppression of T cell activation in patients with chronic hepatitis B

**DOI:** 10.1186/s12865-015-0088-x

**Published:** 2015-05-16

**Authors:** Ying Chen, Zuxiong Huang, Di Ma, Liqian Chen, Qintao Lai, Xuan Huang, Jia Zhou, Xiaoyong Zhang, Qiang Ma, Zhengliang Chen, Daming Zuo

**Affiliations:** Department of Immunology, School of Basic Medicine, Southern Medical University, Guangzhou, 510515 China; State Key Laboratory of Organ Failure Research, Guangdong Provincial Key Laboratory of Viral Hepatitis Research, Department of Infectious Diseases, Nanfang Hospital, Southern Medical University, Guangzhou, China; Department of Hepatology, Affiliated Infectious Disease Hospital of Fujian Medical University, Fuzhou, China; Institute of Antibody Engineering, School of Biotechnology, Southern Medical University, Guangzhou, China

**Keywords:** Scavenger receptor A; Chronic hepatitis B; T cell activation;

## Abstract

**Background:**

Scavenger receptor A (SRA) is expressed predominantly in phagocytic cells playing an essential role in the host immune defense against invading microorganisms. Our previous study reported the presence of SRA in a soluble form in patients with infection of hepatitis B viruses (HBV). However, the association of soluble SRA with stages of HBV infection and the immune response induced by HBV is not fully determined.

**Methods:**

In this study, we detected soluble SRA in serum from 29 chronic hepatitis B (CHB) patients, 28 chronic HBV carriers in the immune tolerant (IT) stage, 33 in the HBeAg-negative inactive carrier (IC) stage, and 22 healthy controls (HCs), respectively. We further analyzed the correlation of detected soluble SRA to inflammation and serum viral load. In addition, we investigated the regulatory role of soluble SRA in T cell activation, especially in CD8^+^ T cell response to HBV peptide.

**Results:**

We demonstrated that Median levels of serum soluble SRA in CHB and IT patients were significantly higher than those of IC patients and HCs. Additionally, the concentrations of soluble SRA were negatively correlated with alanine transaminase levels in CHB patients. We also found that serum concentration of SRA was decreased during telbivudine treatment. Expressed SRA extracellular domain suppressed HBV core peptide-stimulated interferon-γ and tumor necrosis factor-α production in CD8^+^ T cells, and it bound to T cells in a higher frequency in CHB patients than in HCs. Furthermore, we observed that naïve human T cells stimulated by anti-CD3 and CD28 antibodies in the presence of the recombinant SRA protein had reduced activation and proliferation.

**Conclusion:**

In summary, we determined the level of soluble SRA in different stages of CHB patients. SRA might inhibit T cell proliferation and activation as a soluble form. These results not only revealed a previously unknown feature of soluble SRA in CHB patients but also provided broad understanding of SRA in T cell activation.

**Electronic supplementary material:**

The online version of this article (doi:10.1186/s12865-015-0088-x) contains supplementary material, which is available to authorized users.

## Background

Hepatitis B is a serious and potentially life-threatening liver infection caused by the hepatitis B virus (HBV), which has become a major global health problem. About two billion people worldwide have been infected with the virus, and more than 350 million have chronic (long-term) infection [1]. Chronic infection with HBV can lead to severe liver diseases including advanced fibrosis, cirrhosis, and hepatocellular carcinoma (HCC), causing an estimated 600,000 deaths each year [2]. In patients with chronic HBV infection, there is a reduction or functional exhaustion of HBV-specific CD4^+^ and CD8^+^ T cell responses compared with responses from individuals who succeed in resolving infection [3,4]. CD8^+^ T cells, the main effector cells in viral clearance of HBV, produce an array of cytokines, among which interferon gamma (IFN-γ) and tumor necrosis factor alpha (TNF-α) are responsible for inhibition of HBV replication in target cells [5,6]. Exhaustion of CD8^+^ T cells has been demonstrated in patients with chronic HBV infection [7]. Lopes *et al.* found pro-apoptotic protein Bcl2-interacting mediator (Bim) was upregulated in HBV-specific CD8^+^ T cells from patients with chronic infections compared with resolved infections [8,9]. In addition, increased expression of inhibitory molecules such as PD-1, CTLA-4, and TIM-3 may contribute to dysfunction and apoptosis of virus-specific CD8^+^ T cells [9-12]. Other extrinsic factors in the liver microenvironment, such as immunosuppressive cytokines IL-10 and TGF-β can also hamper the ability of T cells to expand and survive, thereby attenuating anti-viral control [13]. Thus, blockade of the inhibitory pathways could be a logical, reasonable therapeutic strategy to rescue dysfunctional T cells and would likely restore functional T cell response in patients.

Scavenger receptor A (SRA, also called CD204) is expressed primarily on phagocytic cells or antigen presenting cells (APCs), such as dendritic cells (DCs) and macrophages [14], as well as on liver sinusoidal endothelial cells (LSECs) [15]. SRA has been studied extensively in the context of atherosclerosis or cardiovascular diseases, where it was identified initially as a major receptor for internalization of modified lipoproteins [16]. SRA has also been shown to function as an innate pattern recognition receptor (PRR) capable of recognizing a broad spectrum of “self” and “non-self” ligands, including modified or altered molecules, pathogen-associated molecules, and endogenous danger molecules such as stress proteins [14,17]. Studies emerging from the field of tumor immunology showed SRA functions as a suppressor of T-cell activation and antitumor immunity [18,19]. SRA also suppresses CTL and Th1 responses triggered by model antigen OVA with LPS or monophosphoryl lipid A (MPL), a pathogen-associated molecular pattern (PAMP) that engages the toll-like receptor 4 (TLR4) signaling pathways [20,21]. It has been reported that SRA is responsible for uptake of adenovirus 5 in macrophages [22]. However, little is known about the role of SRA in the pathogenesis of chronic HBV infection and the virus-induced T cell response.

Our previous study found increased serum levels of soluble SRA in patients were associated with occurrence of chronic hepatitis B (CHB) infection [23]. In the present study, we conducted a cross-sectional comparison of the levels of soluble SRA in subjects who were either HBeAg-positive or HBeAg-negative, and we evaluated the concentration of serum SRA in subjects of telbivudine treatment. We analyzed the effect of SRA on *in vitro* activation of HBV peptide-induced CD8^+^ T cell or anti-CD3/CD28-induced T cell activation. In addition, we investigated the interaction between soluble SRA and T cells and the effect of SRA on T cell priming. Our study revealed SRA negatively regulated HBV-induced CD8^+^ T cell responses in a soluble form, which might represent a mechanism of T cell exhaustion in CHB patients.

## Results

### Elevated levels of serum-soluble SRA in CHB patients

**Figure 1:**
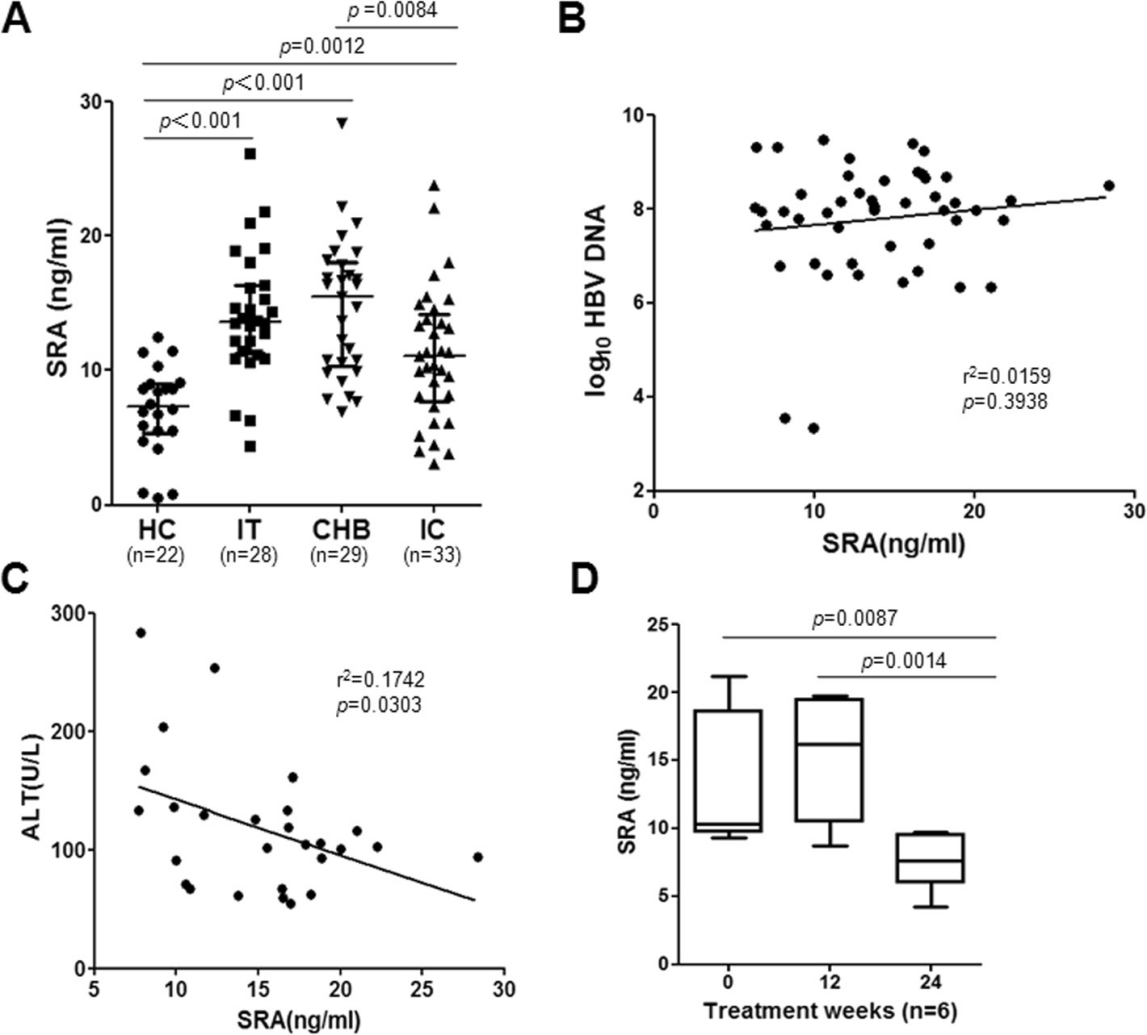
Cross-sectional analysis of the soluble SRA level in patients with hepatitis B virus. **(A)** Comparison of serum soluble SRA concentrations in healthy controls (HC) (n = 22), subjects in the immune tolerance (IT) (n = 28) and inactive carrier (IC) (n = 33) stages of a chronic HBV infection, and patients with HBeAg-positive chronic hepatitis B (CHB) (n = 29). **(B)** The relationship of serum soluble SRA concentration with HBV DNA in patients with HBV DNA above 1000 copies/mL (n = 48). **(C)** The relationship of serum soluble SRA concentration in CHB patients (n = 29) with alanine transaminase (ALT) levels. **(D)** Serum SRA concentrations at baseline and at 12 and 24 weeks after starting telbivudine therapy in 6 patients with HBeAg-positive chronic hepatitis B.

Our earlier observation of increased levels of soluble SRA in mice with immune-mediated liver injury and clinical hepatitis [23] prompted us to evaluate the association between stages of chronic HBV infection and serum soluble SRA concentrations in this cross-sectional study (Table 1). We evaluated the concentration of soluble SRA in serum from patients and HCs using ELISA. Soluble SRA levels in CHB patients were significantly higher than those in HCs and IC patients. In contrast, there was no significant difference in soluble SRA concentrations between CHB patients and IT patients (Figure 1A).

**Table 1:** **Clinical characteristics of subjects in cross sectional study**

**Group**	**HC**	**IT**	**CHB**	**IC**
No. of patients	22	28	29	33
Gender(M/F)	12/10	10/18	18/11	19/14
Age(year)	25.0 (22.2-33.8)	26.0 (17.4-36.2)	27.0 (22.0-38.0)	33.0 (22.0-43.8)
HBV-DNA(log_10_ copies/mL)	n.d.	8.00 (6.41-9.30)	7.98 (6.57-9.02)	<3
ALT(U/L)	14.5 (8.3-27.9)	26.0(15.6-35.0)	104.2(60.6-214.0)	19(12–30.8)
HBsAg positive	0	28	29	33
Anti-HBs positive	16	0	0	0
HBeAg positive	0	28	29	0
Anti-HBe positive	0	0	0	33
Anti-HBc positive	0	28	29	33

Data are shown as median (10–90% percentile).

ALT, alanine aminotransferase; CHB, chronic hepatitis B; HC, healthy control; IC, inactive carrier; IT, immune tolerant;

HBsAg, hepatitis B surface (HBs) antigen; HBeAg, hepatitis B e antigen; n.d., not determined.

We analyzed the correlation of serum SRA level and HBV DNA load in patients with HBV DNA above 1000 copies/mL (n = 48), finding no significant correlation between the level of soluble SRA and plasma HBV DNA load (Figure 1B). We next analyzed the correlation between serum SRA level and alanine aminotransferase (ALT) level in the CHB patients (n = 29). In contrast to the results for HBV DNA load, concentration of soluble SRA was negatively correlated with ALT level (Figure 1C). Additionally, the serum level of SRA was decreased significantly after telbivudine treatment (Figure 1D and Table 2).

**Table 2:** **Clinical characteristics of the subjects with telbivudine therapy (n = 6)**

**Treatment week**			
Variable	0	12	24
HBV-DNA(log_10_ copies/mL)	8.64 (6.84-9.18)	5.06 (3.30-6.09)	3.86 (2.46-5.38)
ALT(U/L)	63.0 (15.0-147.0)	31.0 (21.0-79.0)	29.0 (17.0-51.0)
TBil (μmol/L)	14.4 (5.5-14.9)	14.8 (7.7-27.7)	18.4 (6.5-25.4)
SRA(ng/mL)	10.4 (9.3-21.2)	16.19 (8.7-19.8)	7.6 (4.2-9.7)

Data are shown as median (10–90% percentile). TBil, total serum bilirubin.

### Extracellular domain of SRA suppressed HBV antigen-induced CD8^+^ T cell response

**Figure 2:**
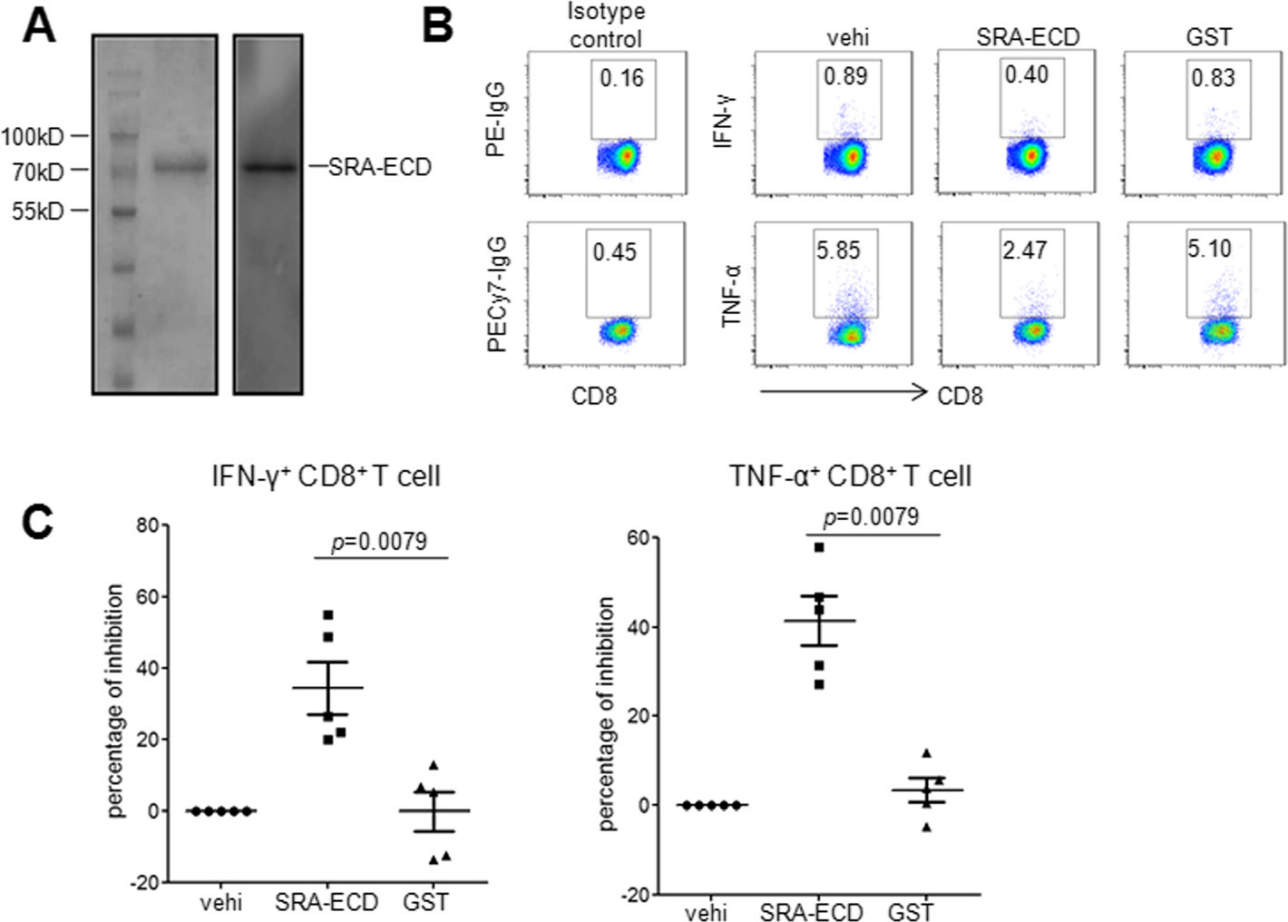
SRA-ECD protein suppressed HBV antigen induced CD8^+^ T cell responses. **(A)** Recombinant SRA-ECD protein was prepared using the pET expression system, then analyzed by SDS-PAGE and immunoblotting with anti-SRA antibody. **(B)** PBMCs from CHB patients were stimulated with HBV core peptides, and subjected to intracellular cytokine staining assays. The percentages of HBV peptide-induced cytokine-producing CD8^+^ T cells were calculated. Representative dot plots from three independent experiments are shown. GST protein prepared by the same procedure was used as an unrelated protein control. The inhibited percentages were calculated and summarized **(C)**.

To further observe if there is any potential effect of soluble SRA on HBV-induced CD8^+^ T cell response in chronic HBV infection, we prepared a recombinant extracellular domain of the SRA protein (SRA-ECD) using the pET expression system (Figure 2A). The extracellular collagenous domain of SRA has been known to bind lipopolysaccharide (LPS) [24], and the recombinant protein has similar ligand-binding property as endogenous SRA as determined by ELISA (Addtional file 1 Figure 1). SRA-ECD protein significantly inhibited the production of cytokine IFN-γ and TNF-α by CD8^+^ T cells stimulated with HBV-specific peptides (Figure 2B and C). To rule out the possibility that the suppressive effect might be artificially derived from the expression system, we used a GST tag expressed by the identical prokaryotic expression vector as a negative control. We found that the expression system itself had no effect on HBV antigen-induced CD8^+^ T cell function (Figure 2B). Compared with the vehicle group treated with HBV peptide stimulation at 0%, the reduction of IFN-γ and TNF-α in the SRA-ECD protein-treated group were statistically significant (Figure 2C).

**Figure 3:**
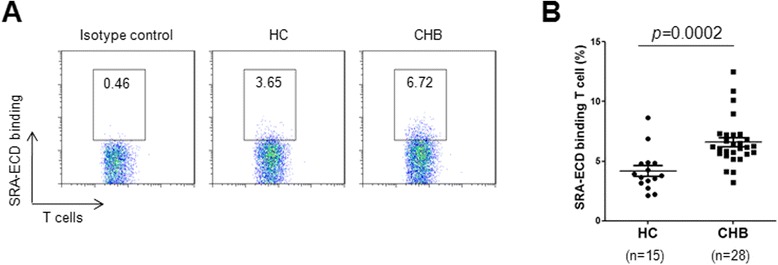
SRA-ECD protein binds to T cells from CHB patients more efficiently. Whole blood from healthy controls and CHB patients was analyzed for SRA-ECD binding with antibodies for CD3 after red blood cell lysis **(A)**. Summaries of 15 healthy controls and 28 CHB patients are shown **(B)**.

We next assessed the occurrence of SRA binding to T cells during the HBV-induced immune response. We employed a biotinylated recombinant SRA-ECD protein to target a putative receptor on T cells, and found SRA-ECD bound a subset of T cells (Figure 3A). Strikingly, the percentage of positive T cells was significantly higher in CHB patients than in healthy controls (Figure 3B).

### SRA-ECD protein inhibited T cell proliferation and cytokine production

**Figure 4:**
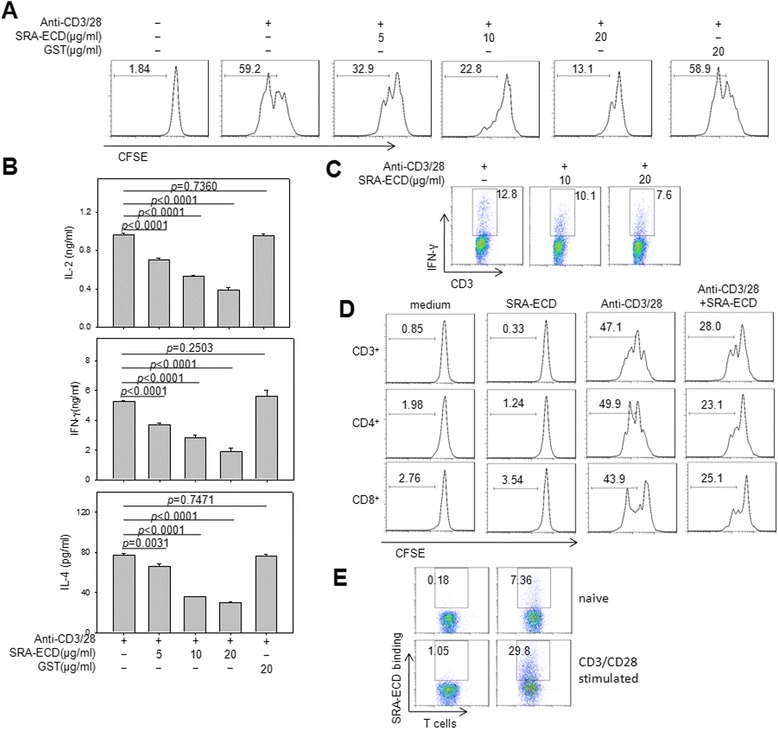
SRA-ECD protein inhibits T cell proliferation and cytokine production. **(A, B)** T cells purified from human PBMCs were stimulated with anti-CD3 (1 μg/mL) and anti-CD28 (1 μg/mL) antibodies in the presence of the indicated concentration of SRA-ECD protein for 72 hr. T cell proliferation was assessed by FACS analysis of CD3^+^ T cells based on the dilution of CFSE intensity. Histograms are representative of two independent experiments **(A)**. Supernatants were collected after 48 h and assayed for IL-2, IFN-γ and IL-4 using ELISA **(B)**. **(C)** Naïve T cells stimulated with anti-CD3 and anti-CD28 antibodies for 72 hr. Then the cells were re-stimulated with anti-CD3 and anti-CD28 antibodies in the absence (medium) or presence of different concentration of SRA-ECD protein for 6 hours and subjected to intracellular cytokine staining assays. The percentages of IFN-γ-producing CD3^+^ T cells were calculated. Representative dot plots from two independent experiments are shown. **(D)** CFSE-labeled naïve T cells were stimulated in plates coated with anti-CD3 anxd anti-CD28 antibodies in the absence or presence of 10 μg/mL of SRA-ECD protein. After 72 h, T cells were stained for CD3, CD4 and CD8 respectively. T-cell proliferation was assessed by FACS analysis based on the dilution of CFSE intensity. Histograms are representative of three independent experiments. **(E)** Human PBMCs were activated with anti-CD3 and anti-CD28 antibodies co-stimulation for 48 h, and cells with and without stimulation were analyzed for SRA-ECD binding together with antibodies for CD3. Data are representative of three independent experiments.

To evaluate the function of SRA on T cell receptor (TCR)-mediated T cell activation and function, we stimulated purified T cells from healthy controls with both anti-CD3 and anti-CD28 antibodies in the absence or presence of different doses of SRA-ECD recombinant protein. As expected, SRA-ECD protein inhibited T cell proliferation (Figure 4A) and cytokine (i.e., IL-2, IFN-γ and IL-4) production (Figure 4B) in a dose-dependent manner. In contrast, GST protein did not have any suppressive effect on T cell activation, suggesting the specificity of inhibitory activity of SRA-ECD recombinant protein for T cell priming and activation. To determine whether the T cell suppression function of SRA is a result of SRA-mediated T cell apoptosis or inhibition of T cell activation, we evaluated T cell apoptosis using Annexin V staining after 3 days of stimulation with anti-CD3 and anti-CD28 antibodies stimulation in the presence or absence of recombinant SRA protein. The results showed no difference between the groups treated with SRA-ECD protein versus GST protein (Additional file 1 Figure 2). The intercellular staining of IFN-γ confirmed the suppressive function of soluble SRA on T cell activation (Figure 4C). We also found the inhibitory effect of soluble SRA on T cell activation through T cell receptor engagement was similar between CD4^+^ or CD8^+^ T-cell subsets (Figure 4D).

Furthermore, we measured the binding capacity of SRA to naïve T cells or anti-CD3/CD28 stimulated T cells. Stimulated T cells were strongly bound by SRA-ECD protein compared with naïve T cells (Figure 4E).

### SRA-ECD inhibition of T cell proliferation was IL-2-dependent and involved in initiating TCR signal transduction

**Figure 5:**
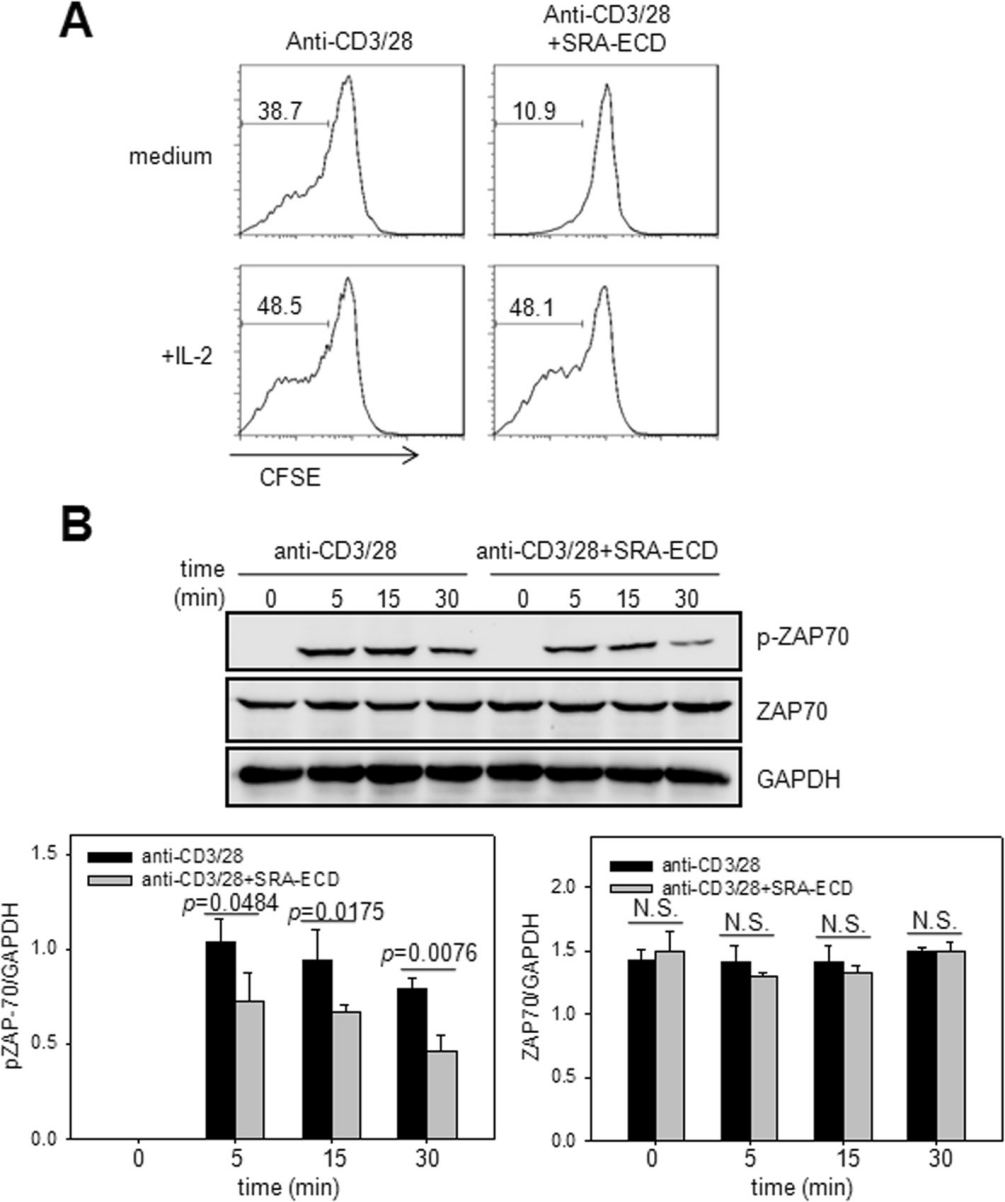
Impaired T proliferation by SRA-ECD could be reverted by IL-2. **(A)** CFSE-labeled naïve T cells were stimulated with immobilized anti-CD3 (0.5 μg/mL) and anti-CD28 (0.5 μg/mL) antibodies or anti-CD3/28 plus SRA-ECD in the presence or absence of recombinant IL-2 (100 U/mL). After 72 h, cells were stained for CD3, and proliferation and cell death induction was determined for CD3^+^ T cells. The data are representative of one experiment among three independent experiments. **(B)** Purified T cells were activated on plates coated with anti-CD3 and anti-CD28 antibodies in the absence or presence of SRA-ECD protein. Phosphorylation of ZAP-70 was determined by western blot analysis at different time points. GAPDH was used as a loading control. Immunoblot bands were quantified by densitometry analysis using ImageJ software. The data are presented as ratio of protein expression compared with GAPDH expression for each sample. The result presented is from one representative experiment among two independent experiments. N.S. stands for not statistically significant.

To further investigate whether the inhibition of T cell activation by soluble SRA-ECD protein was the result of a decrease in IL-2, we added a high concentration of exogenous IL-2 to the T cells treated with anti-CD3/CD28 co-stimulation in the presence or absence of SRA-ECD protein. Complementary IL-2 could restore proliferation of T cells in the presence of both SRA-ECD protein and anti-CD3/CD28 (Figure 5A). No effect of exogenous IL-2 was observed in proliferation of T cells without stimulation in absence or presence of SRA-ECD protein (data not shown). Taken together, these results implied that inhibition of T cell activation in the presence of SRA-ECD protein might be due to reduced expression of IL-2.

We next tested whether soluble SRA might interfere with early signaling events after TCR engagement. Considering that TCR signal transduction requires activation of ZAP-70 protein tyrosine kinase for all downstream signaling events [25], we evaluated the effect of SRA protein on the ZAP-70 phosphorylation upon T cell activation. Western blot analysis showed that the presence of SRA-ECD protein during initiation of TCR signal transduction was associated with inhibition of phosphorylation of ZAP-70 (Figure 5B).

## Discussion

We previously identified the presence of a high level of soluble SRA in serum from patients with HBV infection [23]. However, the mechanism underlying this phenomenon remains unclear. The purpose of this study was to validate our hypothesis that soluble SRA could negatively regulate HBV-induced immune effector functions, thereby impeding the host response towards HBV eradication. We first conducted a cross-sectional study to investigate the association between serum levels of soluble SRA and the clinical status of subjects with HBV infection. We next demonstrated the inhibitory effects of soluble SRA on HBV peptide-induced CD8^+^ T cell responses and analyzed the interaction between SRA and T cells. Our studies provided a preliminary understanding of the involvement of soluble SRA during HBV infection and its action in modulating the antiviral immunity.

Few studies have previously examined the role of SRA in viral infection and immunity; however, uptake and degradation of adenoviruses through SRA by macrophages has been reported [22]. Here, we observed that serum SRA levels in CHB patients were comparable to that in HBeAg-positive immunotolerant patients, but were significantly higher than those of the inactive carriers who had completed HBeAg seroconversion. It was previously reported that functional responsiveness of circulating HBV-specific CD8^+^ T cells was crucial for eliciting an HBeAg response [26,27]. This raised the possibility that higher levels of soluble SRA might be associated with suppression of anti-viral CD8^+^ T cell responses, which could subsequently cause persistent HBV infection and failure of HBeAg seroconversion while reducing SRA concentration in patients. This might favor restoration of anti-HBV CD8^+^ T cell responses during anti-viral treatment. Among patients with CHB, correlation between serum ALT and soluble SRA levels implied that the serum levels of SRA might be biologically related to the severity of liver inflammation. This indicated that soluble SRA might be an inflammatory biomarker upregulated in the setting of CHB and downregulated with treatment, but does not necessarily impact viral clearance. It should be noted that telbivudine treatment can significantly decrease the HBV DNA load in the CHB patients [28]. In our study, we also found a decrease of serum SRA levels after telbivudine treatment. SRA protein might also participate in the suppression of chronic viremia, which remains to be investigated. Yla¨-Herttuala *et al*. derived soluble SRA-containing medium from AAVsMSR-transduced rabbit fibroblasts, and they found that the conditioned medium both inhibited foam cell formation *in vitro* [29] and suppressed the THP-1 monocyte/macrophage adhesion on OxLDL-activated endothelial cells [30]. These results indicated that soluble SRA might influence atherogenesis and the subsequent inflammatory response. Indeed, there were a number of studies showing a regulatory function for cell-surface SRA in the inflammatory response. Kobzik *et al*. reported that SRA suppressed production of IL-12, a crucial regulatory cytokine driving the Th1 response, in macrophages stimulated with CpG or LPS plus IFN-γ [31,32]. A previous study showing that SRA^−/−^ mice produced more IL-10 compared with wild-type mice emphasized the involvement of SRA in the anti-inflammatory response [33]. We speculated that soluble SRA in circulation might limit the inflammatory response inside liver the as it does in the peripheral system.

CD8^+^ T cell deletion experiments performed in HBV-infected chimpanzees have provided strong support for the concept that CD8^+^ T cells are the main cellular subset responsible for viral clearance [6]. It has been reported that responses with a Th1 profile of cytokine production by HBV-mediated CD4^+^ and CD8^+^ T cells were associated with a more favorable disease outcome [6,34]. Patients with chronic HBV infection have a weak or undetectable virus-specific T-cell response [35,36]. A growing body of evidence revealed the role of SRA in suppressing antigen-specific CTL responses against tumors [18]. The effect of SRA on CD8^+^ T cell responses was also examined using the model antigen OVA. The result showed that SRA^−/−^ DCs were much more effective than wild-type DCs in driving OT-I cell proliferation *in vitro* [20]. In addition, we previously found that the percentage of IFN-γ-producing CD4^+^ T cells in SRA^−/−^ mice was significantly higher compared with those in WT mice after vaccination with OVA immunogen [21]. We have shown that soluble SRA protein suppressed HBV peptide-induced CD8^+^ T cell response compared with an unrelated control protein (GST). The data suggested the soluble form of SRA, like its membrane-bound counterpart, exhibits a direct suppressive effect on T cell response. Surprisingly, we found that SRA protein also reduced the amount of IL-4 secretion upon T cell activation. IL-4 is the primary cytokine implicated in the development of Th2-mediated responses, which is associated with allergy and asthma [37]. A previous study found that SRA deficient mice showed a much more allergic inflammatory response compared to the wild-type counterpart [38]. It is worthy to investigate whether and how SRA regulates Th2 response during asthma.

The SRA-ECD protein we prepared had higher efficiency binding to T cells from patients with HBV infection than from healthy controls. Further study revealed that SRA-ECD protein preferably interacted with activated T cells rather than naïve T cells. This indicated soluble SRA might recognize unidentified receptors mainly induced after T cell activation. TCR engagement by antigens triggers a cascade of tyrosine phosphorylation of signaling components, leading to the activation of multiple pathways, which ultimately induces T cell growth factor (e.g. IL-2) gene expression. When the TCR interacts with peptide antigen bound to a MHC complex molecule on antigen presenting cells, the TCR co-receptor CD4 or CD8 binds to the MHC complex, activating the co-receptor associated tyrosine kinase Lck, which is brought into proximity of the CD3 complex and phosphorylates tyrosines in the immunoreceptor tyrosine-based activation motifs (ITAMs). When doubly phosphorylated, ITAMs interact with the tandem SH2 domains of ZAP-70. Active ZAP-70 subsequently phosphorylates LAT and SLP-76, which recruit many other signaling molecules and lead to T-cell activation, proliferation, and differentiation [25]. Subsequently, IL-2 binds its receptors to promote activated T cell proliferation with nucleotide synthesis through the PI3K/AKT pathway and mammalian target of rapamycin (mTOR)/p70S6K pathway. Our study showed that the inhibitory effect of SRA-ECD protein on T cell activation could be through an IL-2 dependent manner. SRA-ECD protein greatly reduced the IL-2 production and addition of exogenous IL-2 restored the proliferation of T cells treated with SRA-ECD protein. These results demonstrated that soluble SRA could serve as a negative regulator of T cell activation. However, it remains to be determined how soluble SRA regulates the initiation of T cell signal transduction.

## Conclusion

Taken together, increased soluble SRA expression in patients with chronic HBV infection is important for decreasing T cell activation, and soluble SRA is involved in inflammatory liver pathology. This study demonstrated soluble SRA served as a negative regulator in T cell responses. Compared with recently identified co-inhibitory molecules (e.g., PD-1, CTLA-4, TIM-3) present on T cells [9-12], our research is focused on a host-derived inhibitory molecule that is mainly expressed on myeloid cells or present in a soluble form. Insightful understanding of the negative regulation of SRA in inflammation and immunity in HBV infection may provide new opportunities for therapeutic intervention in CHB patients.

## Methods

### Human Subjects

Twenty-nine chronic hepatitis B (CHB) patients, 28 chronic HBV-infected patients in the immune tolerant (IT) stage, 33 in the HBeAg-negative inactive carrier (IC) and 22 healthy controls (HC) were enrolled in the cross-sectional study. CHB patients as well as IT and IC were diagnosed according to the described criteria [39]. Subjects with previous antiviral therapy, co-infection of HIV or other hepatitis virus, diabetes, severe systemic illness, regular alcohol over-consumption and hepatocellular carcinoma were excluded. Basic characteristics of the enrolled subjects were summarized in Table 1.

Six CHB patients from a phase IV, multi-center, open-label clinical trial of telbivudine (600 mg/d, n = 21, trial number CLDT600ACN07T) were enrolled for longitudinal study, and heparinized blood was taken at baseline, 12 and 24 weeks of telbivudine treatment (Table 2), respectively. The study protocol was approved by the Medical Ethical Committee of Nanfang Hospital of Southern Medical University. Prior to the collection of blood sample, the informed consent for participation in the study was obtained from each participant or parent if the participant is under 18 years of age.

### Serological assays and HBV-DNA assays

Presence of HBsAg, HBeAg, anti-HBs, anti-HBc, anti-HBe, anti-HCV, and anti-HDV was determined using commercial AxSYM MEI kits (Abbott Laboratories, North Chicago, IL, USA). The HBV-DNA level was quantified using the Roche Diagnostics Cobas Taqman 48 (Meylan, France). The biochemical analyses were conducted with an OLYMPUS AU5400 Full Automatic Biochemical Analyzer (Olympus Corp., Tokyo, Japan).

### ELISA Analysis of Soluble SRA

Soluble SRA levels in human serum samples were measured in duplicate using a commercial ELISA kit purchased from Uscn Life Sciences (Wuhan, China).

### Protein preparation

Recombinant extracellular domain of human SRA was expressed in *Escherichia coli* using the pET expression system (Novagen, Madison, WI, USA) and purified by nickel-chelating resins (GE Healthcare, Piscataway, NJ, USA) according to the protocols. The expressed protein was determined by sodium dodecyl sulfate-polyacrylamide gel electrophoresis (SDS-PAGE) analysis and western blot analysis using anti-human SRA antibody (Abcam, Cambridge, MA, USA).

### Cell surface and intracellular staining

Whole blood was incubated with FCM Lysing Solution (Mutisciences, Hangzhou, China) for removal of red blood cells, followed by staining with SRA-ECD protein biotinylated with Sulfo-NHS-LC-Biotin (Pierce, Rockford, IL, USA) and anti-CD3-APC (BD Biosciences, San Jose, CA, USA) for flow cytometric analysis of binding of SRA-ECD protein with T cells.

Intracellular cytokine staining (ICS) was performed as previously described [40,41]. Briefly, freshly isolated PBMCs from CHB patients and HLA-A2(+) PBMCs were selected for stimulation by HBV core18-27 peptide (FLPSDFFPSV) and 18-mer overlapping peptide pool covered C open reading frame (1 μg/mL, GL Biochem, Shanghai, China) plus anti-CD49d (1 μg/mL, BD Biosciences) and anti-CD28 (1 μg/mL) for 10 days. Then the cells were re-stimulated with 3 μg/mL of core18-27 and the 18-mer peptide pool for 1 hour, followed by incubated with 1 mg/mL of brefeldin A (BD Biosciences) for an additional 5 hours. After surface staining with anti-CD8-APC (BD Biosciences), cells were fixed, permeabilized, and stained with anti-IFN-γ-PE and anti-TNF-α-PECy7 (BD Biosciences) respectively according to the manufacturer’ s instructions. In this study, positive response was defined as greater production of either IFN-γ or TNF-α by core peptide-stimulated CD8^+^ T cells compared with either culture medium-stimulated or HIV gag peptide-stimulated CD8^+^ T cells. Patients with a positive CD8^+^ T cell response were chosen for the T-cell suppression assays. The percent inhibition was calculated by the following formula: percentage of inhibition = [(normal activity - inhibited activity)/(normal activity)] ×100%.

### In Vitro T-Cell Stimulation

CellTrace 5(6)-carboxyfluorescein diacetate succinimidyl ester (CFSE) (Molecular Probes, Eugene, OR, USA) labeled human T cells were plated at 2 × 10^5^ cells/well in 1 μg/mL of anti-CD3 Abs (eBioscience, San Diego, CA, USA)-coated plates in the presence of 0.5 μg/mL of anti-CD28 Abs (eBioscience) with or without indicated concentrations of recombinant SRA-ECD protein. In some case, 100 U/ml of IL-2 was added in the incubation. Proliferation was analyzed using FACS based on the dilution of fluorescence intensity. Supernatants were collected at 48 hours after the stimulation for cytokine assays using commercial ELISA kit (eBioscience).

### Western blot

Purified T cells were serum starved overnight in complete medium supplemented with 0.5% FCS, and subsequently activated on anti-CD3 antibodies (1 μg/ml) coated plates in the presence of anti-CD28 antibodies (1 μg/ml) with or without SRA-ECD protein (10 μg/ml). Activated cells were lysed using RIPA buffer (50 mM Tris, 150 mM NaCl, and 1% Nonidet P-40, pH 7.4) and protein lysates were subjected to western blot analysis with antibodies against phospho-ZAP70, ZAP-70 (Cell Signaling Technology, Danvers, MA, USA) or GAPDH (Thermo scientific Inc., Waltham, MA, USA).

### Statistical analysis

Continuous data are expressed as either the median (10th–90th percentile) or the mean ± SEM. Independent samples t test, Mann–Whitney test or Chi square test were used in group comparisons. Spearman’s rank order correlation coefficient was used for correlation analysis. All statistical analyses were based on two-tailed hypothesis tests with a significance level of *p* < 0.05.

## Additional file

Additional file 1: Supplement Figure 1. Recombinant SRA-ECD protein binds with LPS. **Supplement Figure 2.** Recombinant SRA-ECD protein does not induced T cell apoptosis during anti-CD3/CD28 stimulation.

## Abbreviations

ALT: alanine transaminase
APC: antigen presenting cell
Bim: Bcl2-interacting mediator
CD: clusters of differentiation
CFSE: 5-(6)-carboxyfluorescein diacetate, succinimidyl ester
CHB: chronic hepatitis B
CTL: cytotoxic T-lymphocyte
DC: dendritic cell
ELISA: enzyme-linked immunosorbent assay
ECD: extracellular domain
HBV: hepatitis B virus
HC: healthy controls
HCC: hepatocellular carcinoma
IC: inactive carrier
IFN-γ: interferon gamma
IL: interleukin
IT: immune tolerant
LSEC: liver sinusoidal endothelial cell
MAPK: Mitogen-activated protein kinase
MPL: monophosphoryl lipid A
mTOR: mammalian target of rapamycin
NFAT: Nuclear Factor of Activated T-Cells
PAMP: pathogen-associated molecular pattern
PD-1: programmed death 1
PRR: pattern recognition receptor
SDS-PAGE: sodium dodecyl sulfate-polyacrylamide gel electrophoresis
SRA: scavenger receptor A
TBil: total bilirubin
TLR: toll-like receptor
TNF: tumor necrosis factor
