# Supplementary material for: Involvement of soluble scavenger receptor A in suppression of T cell activation in patients with chronic hepatitis B
Source: BMC Immunol. 2015 May 16;16:29. doi: 10.1186/s12865-015-0088-x (PMC4434833; doi:10.1186/s12865-015-0088-x)
Supplement: Additional file 1: Supplement Figure 1. — Recombinant SRA-ECD protein binds with LPS. Supplement Figure 2. Recombinant SRA-ECD protein does not induced T cell apoptosis during anti-CD3/CD28 stimulation. [file 12865_2015_88_MOESM1_ESM.doc]

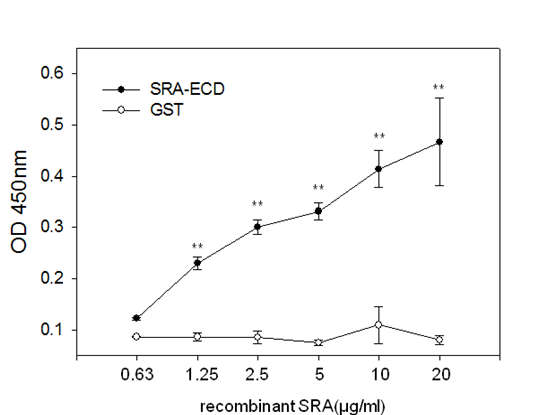


**Supplement Figure 1. Recombinant SRA-ECD protein binds with LPS.** Microtiter wells were coated overnight at 4°C with 20 μg/ml of LPS (Sigma-Aldrich). After blocking, plates were incubated at room temperature for 1 h with different concentrations of SRA-ECD or GST protein. Anti-SRA antibody (Sigma-Aldrich) and anti-GST antibody (Cell Signaling Technology) were added for 1 hour incubation respectively after washing. The levels of bound protein were determined using colorimetric assays after incubation with horseradish peroxidase-conjugated secondary antibody. ** *p*＜0.001. Data are representative of two independent experiments with similar results.


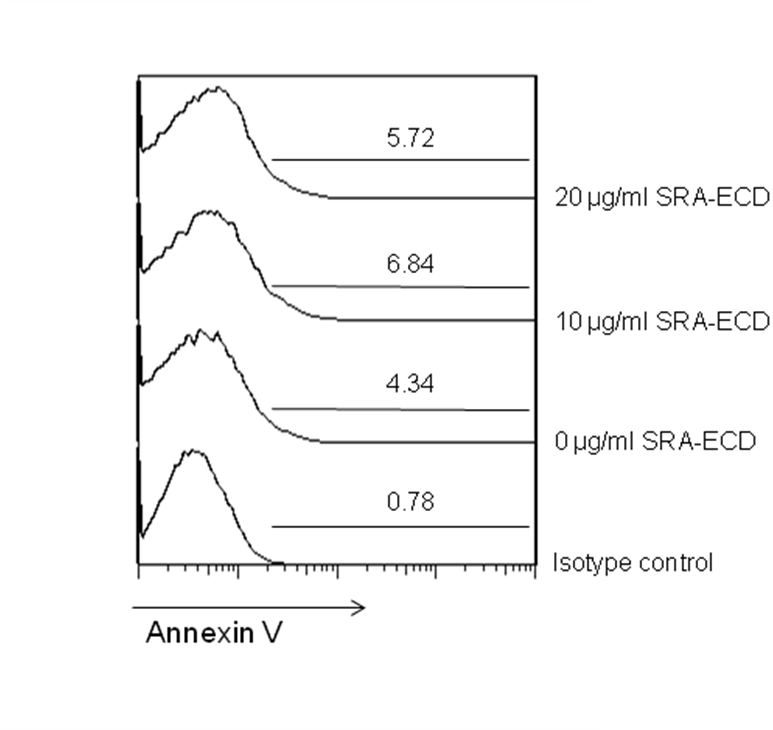


**Supplement Figure 2. Recombinant SRA-ECD protein does not induced T cell apoptosis during anti-CD3/CD28 stimulation.** Naïve T cells were stimulated with anti-CD3 and anti-CD28 antibodies in the absence or presence of different concentrations of SRA-ECD protein for 3 days. The cells were collected for annexin-V staining (KeyGen Biotech, Nanjing, China) according to the manufacturer’s instructions and analyzed using FACS.
